# Automatic 14-plane slice-alignment method for ventricular and valvular analysis in cardiac magnetic resonance imaging

**DOI:** 10.1186/1532-429X-16-S1-P1

**Published:** 2014-01-16

**Authors:** Shuhei Nitta, Taichiro Shiodera, Yukinobu Sakata, Tomoyuki Takeguchi, Shigehide Kuhara, Kenichi Yokoyama, Reiko Ishimura, Toshiya Kariyasu, Masamichi Imai, Toshiaki Nitatori

**Affiliations:** 1 Corporate Research & Development Center, Toshiba Corporation, Kawasaki, Kanagawa, Japan; 2 MRI Systems Division, Toshiba Medical Systems Corporation, Otawara, Tochigi, Japan; 3 Department of Radiology, Kyorin University, Faculty of Medicine, Mitaka, Tokyo, Japan

## Background

Cardiac MRI examinations for valvular heart diseases have recently been a focus of attention [1]. However, the slice-alignment settings for valvular heart diseases are complex, difficult, and time-consuming operations. The purpose of this study is to develop an advanced automatic slice-alignment method that simultaneously detects the six left-ventricular planes (vertical long-axis, horizontal long-axis, short-axis, 4-chamber, 2-chamber, and 3-chamber views), the four right-ventricular planes (short-axis, 4-chamber, 2-chamber, and 3-chamber views), and also the four cardiac valvular planes (LVOT, RVOT, aortic valve, and pulmonary valve views) by extension of a previous work [2]. "'How I do' CMR in valvular heart disease", http://www.scmr.org.

## Methods

ECG-gated 2D steady-state free precession (SSFP) axial multislice cine images covering the entire cardiac region were acquired using a 1.5-T MRI scanner (Excelart VantageTM powered by Atlas, Toshiba Medical Systems) during a single breath-hold with TR/TE = 4.2/2.1, matrix = 198 × 256, number of slices = 16-20, and one image per R-R interval in approximately 20 seconds. The proposed method first detected eight characteristic anatomical features (mitral valve, left-ventricular apex, right-ventricular apex, tricuspid valve, aortic valve, pulmonary valve, left anterior wall of the heart, and right anterior wall of the heart) in an input image using knowledge-based feature recognition and image processing techniques. Next, the directions of the aorta and pulmonary artery were detected based on the distribution of the image gradient around the detected anatomical features. Then, the eight detected positions and the two detected vessel directions were used to determine the positions and orientations of the planes. Finally, for evaluation of the detection results, the normal vector for each view was determined, and the angular error between the detection result and manual annotation was measured for each normal vector.

## Results

**Figure 1:**
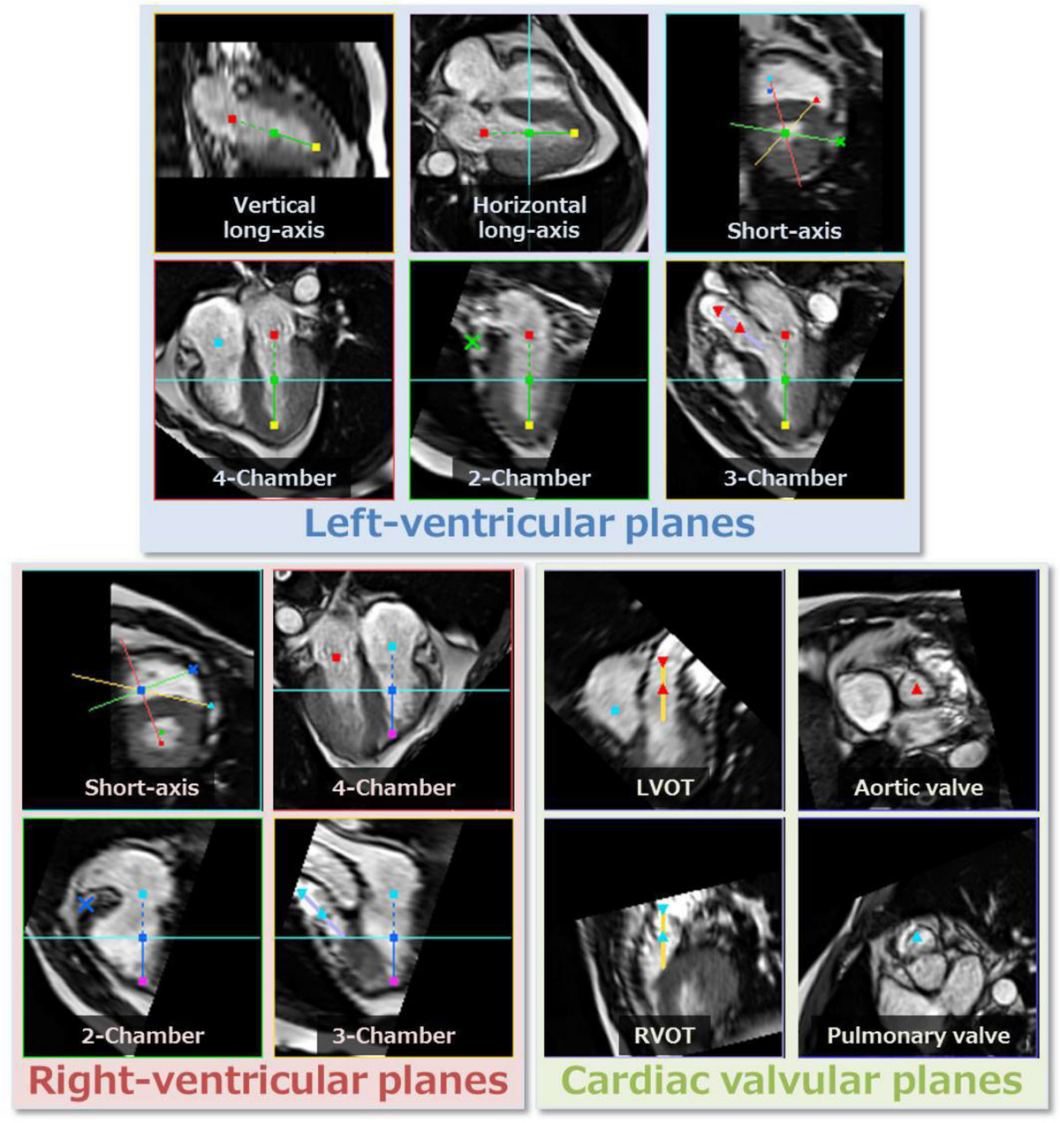
**Example of the detected planes for a healthy volunteer**.

**Figure 2:**
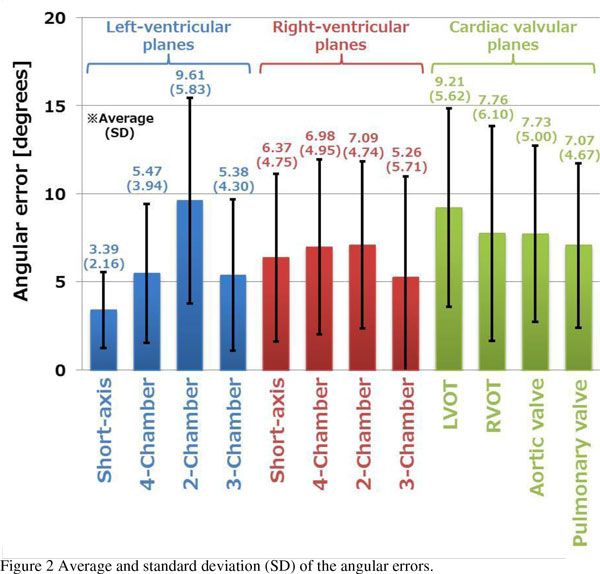
**Average and standard deviation (SD) of the angular errors**.

The proposed method was used for 55 datasets from 23 healthy volunteers, and there were no datasets for which the cardiac planes were undetectable. An example of the detected planes is shown in Figure 1, while the average and standard deviation of the angular errors are shown in Figure 2. The processing time was about 2.5 seconds on a 3.0-GHz CPU.

## Conclusions

The proposed method can detect fourteen cardiac planes in total, including the cardiac valvular planes. The method is clinically useful in various cardiac MRI examinations.

## Funding

No funding was received for this research.
